# The Effect of Freedom upon the Mental and Physical Health of the Negro of the South

**Published:** 1897-01

**Authors:** 


### EDITORIAL.

The office of the Business Manager of The Journal is Nos. 311 and 312 Fitten Building.

The editors are not responsible for views expressed by contributors.

Articles for publication should reach this office not later than the 15th inst.

Address all communications, and make all remittances payable to The Atlanta Medical and Surgical Journal, Atlanta, Ga.

Reprints of articles will be furnished, when desired, at a nominal cost. Requests for the same should always be made *on the manuscript*.

If a subscriber notifies us to stop his Journal and it is not done, or, if he orders his address changed and it is not, he may know that we did not get his order. Moving to another address without notifying us, does not excuse him from paying his bill.

### THE EFFECT OF FREEDOM UPON THE MENTAL AND PHYSICAL HEALTH OF THE NEGRO OF THE SOUTH.

Two important papers have just been published on this subject. ("What has Emancipation done for the Mental and Physical Health of the Negro of the South?" By Dr. J. F. Miller, Superintendent of the North Carolina Colored Insane Hospital. *North Carolina Medical Journal,* November 10, 1896. "The Increase of Insanity and Tuberculosis in the Southern Negro since 1860." By Dr. T. O. Powell, Superintendent of the Georgia Insane Asylum. *Journal of the American Medical Association,* December 5, 1896.)

Each author approaches the consideration of this subject from a wide acquaintance and extensive experience with what may be called the medical side of the negro question, and they have sought to present only facts as they are, without political sentiment or prejudice. Their papers, agreeing with each other on all points, are deserving of attention.

Before the negroes obtained their freedom, Dr. Miller says, insanity and tuberculosis were rare diseases among them.

Many intelligent people of observation and full acquaintance with the negro say that they never saw a consumptive or crazy negro until late years. But abundant testimony from practicing physicians throughout the South shows that the colored citizen no longer enjoys immunity from these diseases. In North Carolina in 1880 there were one hundred insane negroes; in 1895 there were three hundred and seventy-five.

The following table shows the number of colored insane and the ratio to population throughout the United States (U., S. Census Bureau):

| | | |
|---|---|---|
| 1850 | 638 | 175 per million inhabitants. |
| 1860 | 766 | 169 " " " |
| 1870 | 1,822 | 367 " " " |
| 1880 | 6,157 | 912 " " " |
| 1890 | 6,766 | 880 " " " |

Dr. Babcock, of the South Carolina Insane Asylum, says: "We cannot lose sight of the fact that on the basis of the census as compared with insanity in the whites, mental diseases in the negro have arisen from one-fifth as common in 1850 to one-half as common in 1880 and 1890."

The author's data relative to the extent of tuberculosis among the negroes since the war are obtained principally from the colored insane in asylums, and the testimony of these hospitals seems to be that tuberculosis among the blacks has increased *pari passu* with insanity. It would appear from the records of Southern hospitals for the colored insane that there is some peculiar relation between insanity and tuberculosis. In a number of these hospitals the mortality from tuberculosis in some form or other among the colored insane has varied from twelve to forty per cent. of the total causes of death.

Dr. Powell says that according to the census of 1860 there were 44 insane negroes in the State of Georgia, or one to every 10,584 of population. In 1870 there were 129 insane, or one to every

4,225; in 1880 there were 411, or one to every 1,764; and in 1890 there were 910, or one to every 943 of population. Dr. Powell quotes from a letter of Dr. Hopkins, of Thomasville, who says that in twenty years of practice on the plantations before the war, he saw only three cases of pronounced pulmonary tuberculosis. During the war, in the fortifications about Andersonville, where a large number of negroes were constantly employed, and where a register of all patients was kept, he did not see a case of consumption. Dr. Powell regards alcoholic intemperance and syphilis as important causes of insanity in the modern negro. In ante-bellum days secondary and tertiary syphilis were almost unknown among the plantation negroes. "If one had syphilis, the family physician was sent for, and treatment was continued until the patient was fullly cured. He had to tell from whom it was contracted, and if it was from a neighboring plantation the owner was notified, and the individual infected was subjected to the same treatment." The author believes that insanity and tuberculosis are closely allied. "The sudden outburst of insanity among the colored race of the South came associated hand in hand with tuberculosis."

We close with some conclusions from these papers:

"The rapid increase of insanity and consumption in this race is due to a combination of causes and conditions. This race has developed a highly insane, consumptive, syphilitic and alcoholic constitution, which predisposes them to diseases which formerly they were free from. In this disturbed and unstable condition they seem to be totally unable to resist the slightest exciting causes. They are liable to succumb much more readily than the whites; especially is this true in regard to insanity and consumption. These causes could not have existed prior to 1860 to any large extent, or we would have had the same pathological changes and results that we have now. Up to 1865 it was to the interest of the owners not to allow them to violate the laws of health. Therefore their hygienic surroundings were carefully guarded from their youth. Their lives were regular and systematic, and they were absolutely restrained from all dissipation and excesses, and when sick they had the very best medical attention and nursing until pronounced restored by the physician. Freedom removed all hygienic restraints, and they were no longer obedient to the inexorable laws of health, plunging into all sorts of excesses and vices, and having apparently little control over their appetites and passions. It is very manifest that these morbid tendencies and susceptibilities have been growing for the past thirty years; hence their unstable condition, and susceptibility to and inability to resist attacks of disease."—(Powell.)

"Having shown that under his former manner of life the negro enjoyed a wonderful immunity from brain and lung trouble, I confidently assert that the germs of these troubles came to the same man and race in consequence of his changed environments and the manner of his life which followed.

"In his ignorance of the laws of his being, the functions of citizenship and the responsibilities and duties which freedom imposed, demands were made upon the negro which his intellectual parts were unable to discharge. In his former condition none of these things disturbed his mind. Immediately the restraining influences which had been such conservators of healthfulness of mind and body were removed, thousands left the quiet homes and regular life of the country for crowded and badly ventilated houses of the towns.

"The negro in slavery had no thought for the morrow, wherewithal he should be fed and clothed, nor did the claims of family press upon him to worry and affect his mind; no ambitious hopes stirred his brain as to the possibilities of his future; but 'far from the madding crowd's ignoble strife,' he spent his quiet, humble life in his little log cabin, with his master to care for every want of self and family, in sickness and in health.

"It is an undisputed fact, known to our Southern people, that no race of men ever lived under better hygienic restraints or had governing their lives rules and regulations more conducive to physical health and mental repose. Their habits of life were regular, their food and clothing were substantial and sufficient, as a rule, and the edict of the master kept indoors at night and restrained them from promiscuous sexual indulgence and the baneful influences of the liquor saloon. In sickness he was promptly and properly cared for by physician and nurse. Freedom came to him and a change came over his entire life.

"In the light of the teachings of the last two decades, showing a marvelous increase of insanity among the Afro-Americans, and tyrannized, as I fear they henceforth will be, by an heredity from which they cannot escape, I confidently believe that in the near future the proportion of insanity among the negroes in the South will be as great, if not greater, than among the whites."—(Miller.)

---

WE call attention to the letter of Dr. Geo. M. Gould in this issue, which will interest contributors to medical journals. The spirit manifested by the publishing house there named is narrow and ungenerous and deserves an emphatic rebuke from a liberal and broad-minded profession. The writer of a medical paper which has, perhaps, been prepared with great care, or even expense, has an interest in it which does not cease with its publication; it is submitted to the medical press with the hope that it will secure the widest possible circulation, and medical writers will do well to offer their contributions to any of the other equally well-known publishing concerns which are not conducted upon such commercial and niggardly principles. The position taken by these publishers cannot be defended with a single respectable argument.

